# Treatment of *Neotrombicula* species infestation in cats using a 10% (w/v) fipronil topical spot-on formulation: a pilot study

**DOI:** 10.1177/1098612X17715153

**Published:** 2017-06-19

**Authors:** Marie C Cadiergues, Christelle Navarro, Eloy Castilla-Castaño, Line A Lecru, Charline Pressanti

**Affiliations:** 1 UDEAR, Université de Toulouse, INSERM, ENVT, Toulouse, France; 2 Medical Department, Virbac SA, Carros, France; 3 Dermatology Service, Small Animal Hospital, Université de Toulouse, ENVT, Toulouse, France

## Abstract

**Objectives:**

Few data are available concerning therapeutic aspects of feline trombiculiasis. This study evaluated the efficacy of a 10% w/v fipronil-based spot-on solution in 15 cats with natural *Neotrombicula* species infestation.

**Methods:**

Ten cats received 1 drop per affected site on day (D)0 and D14, with the rest of the 0.5 ml pipette applied on the skin between the shoulders. Five cats served as non-treated controls. Parasite score (0 = absent; 3 = severe, >10 parasites/zone) was assessed on D0, D14 and D28 on all animals. Skin lesions (SCORing Feline Allergic Dermatitis lesion severity scale [SCORFAD]) and investigator pruritus scale (IPS; 0 = cat comfortable, grooming like any normal cat; 4 = cat uncomfortable, pruritic all the time) were assessed on treated cats on the same days. Global assessment of efficacy, tolerance and ease of use (GAS; 1 = very poor; 5 = excellent) was assessed on D28.

**Results:**

All the cats completed the study. Parasite scores of the control cats were maintained throughout the trial (mean ± SD: D0 4 ± 0.7, D14 3.2 ± 1.1 and D28 3.2 ± 0.4). In treated cats, SCORFAD (D0 3.2 ± 5.4, D14 1.1 ± 2.1 [*P* <0.002] and D28 0.5 ± 1.3 [*P* <0.002]), parasite (D0 3.9 ± 1.3, D14 1.2 ± 0.8 [*P* <0.005] and D28 0.4 ± 0.5 [*P* <0.005]) and IPS (D0 1 ± 1.2, D14 0.5 ± 1.1 [*P* <0.05] and D28 0.3 ± 0.7 [*P* <0.05]) scores significantly decreased throughout the trial. On D28, the GAS was 4.2 ± 0.9. There were no adverse effects from treatment.

**Conclusions and relevance:**

The 10% w/v fipronil preparation appeared to be effective, safe and practical in the treatment of localised *Neotrombicula* species infestation in these cats.

## Introduction

*Neotrombicula autumnalis* (Acari: Trombiculidae) mites, also called harvest mites or chigger mites, are considered as the most frequent causative agents of trombiculiasis in people and animals.^1–3^ The larval stage is the sole parasitic stage and individuals become infested from the outdoor environment. Harvest mite life cycle is poorly understood. Larvae have a seasonal activity, mainly between September and November.^4^ However, in some countries, particular favourable climatic conditions may allow more than one complete life cycle in a year and larvae might be found during extra periods.^3^ In cats, along with the presence of firmly attached mites, visible to the naked eye as characteristic orange spots, it is possible to observe erythema, papules and crusts, mostly on the ear margins, face, interdigital spaces and ventral abdomen.^3^ Cats can be highly pruritic; however, some individuals can host high numbers of mites without showing any lesions or signs of discomfort.^3^ Seasonality of the infestation and absence of experimental models may explain, at least partially, the lack of licensed products on the market. Previous studies have shown efficacy when treated with systemic selamectin^5,6^ or fipronil spray directly applied on the infested body areas every 2 weeks.^3,7^

The purpose of the present study was to assess the efficacy of a 10% w/v fipronil-based spot-on solution (Effipro 50 mg spot-on solution for cats; Virbac) in cats naturally infested with *N autumnalis*.

## Materials and methods

Fifteen client-owned cats, living outdoors or with outdoor access, were recruited in the south west of France. Both owner consent and approval from the university’s ethics committee were obtained prior to beginning the study. Inclusion criteria were the presence of *N autumnalis* mites on day 0 (D0) and the absence of change in the lifestyle of the cat for the duration of the study. As trombiculiasis is a seasonal skin disease, five cats living in different households, but in the same geographical areas, were included in parallel and left untreated, serving as sentinels to ensure that the tested population would be exposed to potential re-infestations throughout the study. Control cats were selected with few or no skin lesions. Exclusion criteria included cats younger than 2 months of age or weighing <1 kg, cats with a systemic illness or condition that could deteriorate within the following month, and cats with signs or history of allergic skin diseases. Cats that had received an acaricidal treatment within the previous 6 weeks were also excluded.

The study consisted of three visits. Parasitological and dermatological evaluations were conducted on the day of inclusion (D0), day 14 (D14; ± 2 days) and day 28 (D28; ± 2 days) or closing visit for all treated cats; control cats were only assessed for parasitic score. Each case was evaluated by the same investigator throughout the study. Parasite score (PS) was assessed on each body area with a magnifying lens, on a 0–3 scale (0 = absence; 1 = mild, 1–5 parasites/area; 2 = moderate, 6–10 parasites/area; 3 = severe, >10 parasites/area). Skin lesions were scored according to the SCORing Feline Allergic Dermatitis lesion severity scale (SCORFAD), a scoring system used previously.^8,9^ The SCORFAD is a numerical rating scoring system to assess the severity of excoriations, miliary dermatitis, eosinophilic plaques and self-induced alopecia in each of 10 body regions, resulting in a total lesion score of 0–16 per region. Each lesion type was numerically scored independently on a 0–4 scale (0 = none; 1 = very mild; 2 = mild; 3 = moderate; 4 = severe). The 10 body regions evaluated were the head, neck, dorsal and lateral thorax, rump and tail, flanks, sternum and axilla, abdomen, perineum, forelimbs and paws, hindlimbs and paws. The investigator evaluated the intensity of pruritus on a 0–4 scale (investigator pruritus score [IPS]; 0 = the cat was comfortable, grooming like any normal cat; 1 = the cat was grooming, but it was tolerable and the cat remained calm; 2 = the cat was grooming, but it was generally tolerable; 3 = the cat was grooming quite often; the cat was uncomfortable, nervous or often agitated; 4 = the cat was uncomfortable, grooming all the time) on each visit. Global assessment of efficacy, tolerance and ease of use (GAS; 1 = very poor; 5 = excellent) was assessed on D28 by the owner, independently of the investigator. PS, IPS and GAS have previously been used internally for other clinical studies although not in published studies.

Treatment was applied on D0 and D14 by the investigator. One 0.5 ml Effipro 50 mg spot-on solution for cats (fipronil 50 mg; Virbac) pipette was used per cat on each day of treatment. One drop was applied on each affected site. The rest was applied on the skin, in the neck region, between the shoulders.

PS reduction was calculated at each time point (t) using the arithmetic mean of PS according to the following formula: PS reduction (%) = 100 × (mean day 0 – mean t)/mean day 0. Similarly, SCORFAD reduction and IPS reduction were calculated using the same formula.

Wilcoxon signed-rank tests were used to compare data obtained on D14 and D28 with the baseline obtained on day 0. Significance was defined as *P* <0.05. All statistical analyses were performed using XLSTAT 2017-02 (Addinsoft SARL).

## Results

All 15 cats completed the study. The first cat was included on 4 October and the last one was included on 2 November. There were 10 females and five males aged between 6 months and 12 years. No adverse effects was observed.

Parasite scores (mean ± SD) of the control cats were maintained throughout the study (4 ± 0.7 on D0, 3.2 ± 1.1 on D14 and 3.2 ± 0.4 on D28) allowing interpretation of the results in the treated group.

**Figure 1:**
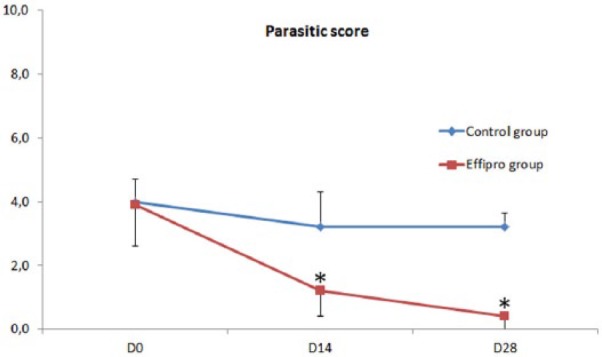
Arithmetic mean ± SD of parasite scores in control cats and cats treated with a 10% w/v fipronil-based spot-on solution. Treatments were applied on D0 and D14. *Significant difference from baseline within a group of cats (*P* <0.05)

On day 0, treated cats had a PS of 3.9 ± 1.3. On D14, it was significantly reduced by 69% (*P* = 0.005); on D28, the PS reduction was 90% (*P* = 0.005). On D28, six cats were parasite-free; the other four had <5 harvest mites on a single body area (Figure 1).

At inclusion, treated cats had a SCORFAD of 3.2 ± 5.4. On D14 it was significantly reduced by 66% (*P* = 0.02); on D28, it was reduced by 84% (*P* = 0.02).

Inclusion IPS was 1 ± 1.2. It was reduced by 50% on day 14 (*P* = 0.04) and further reduced by 70% 2 weeks later (*P* = 0.03).

Owners gave an average GAS of 4.2 ± 0.9 (minimum 2, maximum 5).

## Discussion

This study confirms the efficacy of fipronil against *N autumnalis*, which was previously reported with the spray formulation.^3,7^ To the best of our knowledge, in cats it is the first study with a spot-on solution. In dogs, a line-on solution containing a permethrin–pyriproxyfen combination was tested successfully on nine dogs.^10^ As in the study by Nuttall et al,^7^ we elected to apply the fipronil solution directly on the infested sites. This allowed a higher concentration of the active ingredient in the sites where the parasites are attached. In most cases, parasites were present in the interdigital spaces or on the pinnal margins. After application of a spot-on formulation on the skin between the shoulders, as recommended on the summary of product characteristics, it is expected that the product spreads at the body surface and that the concentration is minimal at the most distanced body regions. Furthermore, these body regions are classically groomed by cats, possibly over-groomed if there is pruritus. Applying the product directly on the parasitised areas ensures a direct efficacy on the parasites already attached and is expected to better prevent re-infestations as mites seem to colonise one or few sites on a given cat.^3^ The presence of the highest concentration of the product where the parasites are supposed to attach is expected to prevent their attachment more rapidly and more efficiently.

The design of the pipette allows a precise, controlled dose to be applied exactly where it is needed. A gentle pressure on the body of the pipette releases the product on the skin; when the pressure stops, the product stays in the pipette without any dripping.

We elected to include a control group to ensure that the cats receiving the treatment would be exposed to harvest mites throughout the study. In the few studies investigating an acaricidal activity against *N autumnalis*, none had a control group.^3,6,7^ As trombiculiasis is a seasonal parasitic disease, in the absence of a control group, the absence of parasites on treated animals could be misinterpreted. In addition to living areas close to those of treated cats, we based the selection of control cats on a level of infestation similar to that of treated cats. However, we found it to be more ethical to only select control cats with few or no skin lesions as they would remain untreated.

The SCORFAD index of treated cats both before and after was low, considering that the maximum possible score is 160. The SCORFAD index is adapted for cats with allergic skin disease. Cats with trombiculiasis are not necessarily allergic, which may explain the low score of cats that were included. This clinical score is helpful as it expresses the clinical improvement, as IPS does. However, they remain secondary outcome criteria in this study and should not be over-interpreted, particularly as included cats were not very pruritic.

The product is licensed in cats for the treatment of flea and tick infestations. The product has a persistent insecticidal efficacy for up to 5 weeks against fleas and a persistent acaricidal efficacy for up to 2 weeks against ticks. In the current study, the product was applied twice at a 2 week interval, based on the approved tick indication in Europe and on published results.^7^ All cats tolerated the two applications well.

## Conclusions

In the cats in the present study, the 10% w/v fipronil preparation appeared to be effective, safe and practical in the treatment of localised *Neotrombicula* species infestation. Recommending the use of the product every 2 weeks from September to November in cats that suffer from trombiculiasis is likely to help in controlling the parasitic disease.
